# Publisher Correction: An immunological autobiography: my year as a COVID-19 vaccine trial participant

**DOI:** 10.1038/s41541-022-00522-9

**Published:** 2022-08-19

**Authors:** Ross M. Kedl

**Affiliations:** Department of Immunology and Microbiology, University of Colorado Anschutz Medical Campus, School of Medicine, Aurora, CO 80045 USA

**Keywords:** Vaccines, RNA vaccines

Correction to: *npj Vaccines* 10.1038/s41541-022-00502-z, published online 18 July 2022

In the original version of this Comment, the ethics declaration was omitted by mistake. The HTML and PDF versions of this Comment have now been updated with the ethics declaration.

